# Stigma and self-esteem across societies: avoiding blanket psychological responses to gay men experiencing homophobia

**DOI:** 10.1192/pb.bp.114.048421

**Published:** 2015-08

**Authors:** Karyofyllis Zervoulis, Evanthia Lyons, Sokratis Dinos

**Affiliations:** 1 BPP University, London; 2 Kingston University, London

## Abstract

**Aims and method** The relationship between homophobia (varying from actual and perceived to internalised) and measures of well-being is well documented. A study in Athens, Greece and London, UK attempted to examine this relationship in two cities with potentially different levels of homophobia. One-hundred and eighty-eight men who have sex with men (MSM) living in London and 173 MSM living in Athens completed a survey investigating their views on their sexuality, perceptions of local homophobia and their identity evaluation in terms of global self-esteem.

**Results** The results confirmed a negative association between homophobia and self-esteem within each city sample. However, Athens MSM, despite perceiving significantly higher levels of local homophobia than London MSM, did not differ on most indicators of internalised homophobia and scored higher on global self-esteem than London MSM. The city context had a significant impact on the relationship.

**Clinical implications** The findings are discussed in relation to the implications they pose for mental health professionals dealing with MSM from communities experiencing variable societal stigmatisation and its effect on a positive sense of self.

Goffman^1^ identified homosexuality as falling into a stigmatised category based on moral failing (i.e. being responsible and therefore being blamed for the condition) and rooted in patriarchal and heteronormative positions as well as the main, mostly monotheistic, religions. The most commonly used term to describe the stigmatisation of lesbian, gay and bisexual (LGB) people is ‘homophobia’ as the dislike or hatred towards homosexuals.^2^ Homophobia is often experienced by gay people from their early years at school, within their families, within their immediate daily environment and in broader society including its institutions, such as the media, workplaces or legislative bodies. Homophobia takes many forms that have been described extensively in the literature in terms of discrimination, violence, prejudice and stereotypes and harassment.^3-6^ Importantly, homophobia appears to affect gay people's own beliefs and attitudes towards themselves. This is known as ‘internalised homophobia’, the phenomenon where gay people are to a variable extent unhappy about their sexual orientation because of societal homophobia.^7^

In general, stigmatisation of an attribute may have a negative effect on a person's positive sense of self. In the case of LGB people, there is evidence that homophobia, both perceived and internalised, affects them in terms of their overall well-being and often in terms of their self-esteem.^8-11^ Self-esteem is an individual's own evaluation of their personal worth and, according to Rosenberg,^12^ it refers to the notion of a relatively stable sense of an overall or global, as it is often described, personal worth. There is a voluminous amount of work which has found a relationship between self-esteem and depression,^13^ anxiety,^14^ anger, hostility and aggressiveness^15^ and life satisfaction.^16^ Therefore, self-esteem is an important construct that mental health professionals need to take into account when dealing with mental health problems.

There are many recent surveys in the UK suggesting that homophobia is still widespread and experienced by gay men in their daily lives, affecting their psychological well-being. A number of studies have also shown the direct relationship between homophobia (or fear of being discriminated against) and mental health problems.^4,6^ Furthermore, recent epidemiological studies found that LGB groups have a significantly higher prevalence of mental health problems than the general population; in particular, conditions including common mental disorders and alcohol and substance misuse, and attempted suicide.^17,18^ In addition to a higher prevalence of mental health problems in gay men and LGB groups in general, further evidence suggests that there are disparities in access to mental healthcare. For example, LGB people report a mixed reception from mental health services.^17^ In particular, mental healthcare for LGB groups is very often associated with depreciation of their domestic circumstances, refusal to accept partners as next of kin, professionals' excessive curiosity about LGB lives, concern about confidentiality, and fear that their sexuality will be regarded as the ‘pathology’ requiring attention.^19,20^ Along these lines, Kitzinger & Coyle^21^ argue that psychological research has always presented homosexuality as a form of pathology and, when dealing with gay or lesbian issues, it is concentrated in clinical and psychotherapeutic psychology.

Societal homophobia is not evident or perceived to be at the same level across all countries or societies. There are historical, cultural and institutional factors that affect its factual or perceived prevalence.^8,21,22^ For example, in institutional terms, there is a broad spectrum that includes countries where homosexual activity is punished to countries where same-sex marriage is recognised. Between these two ends, there are countries where homosexuality is neither illegal nor there are provisions for equal treatment of people irrespective of their sexuality. In relation to the context of this study, there is evidence that Greek society is more homophobic than the UK society^23,24^ and that Greek LGB people tend to face greater difficulties in disclosing their sexuality than Anglo-Americans.^25-27^ In Greece, sex between men was decriminalised in 1951 while there was never any mention of lesbians in Greek law. Today, the age of consent for gay men is set at 17, higher by 2 years than for heterosexuals. There is no official recognition of same-sex couples in any terms. In the UK, although ‘homosexuality’ was decriminalised later, in 1967, legislation today does not discriminate on the basis of sexuality. Currently, same-sex couples can marry and adopt children.

It needs to be noted that metropolitan and cosmopolitan cities such as London consist of people from a variety of countries and cultures. People who live in London or Athens are not exclusively of White English or Greek ethnic background, nor have they been born and lived in either city all their life. So, the term cross-cultural should be avoided in studies such as this one; instead, cross-city comparisons resemble more and should be referred to as cross-national comparisons. The term ‘national’ entails the space and the local rules of law, it does not necessarily refer to participants' nationalities in legal terms and in this case, it can include MSM from potentially any cultural communities that live within the national space, although the existence of a predominant cultural and ethnic majority should be acknowledged.

## Aims

This study aimed to investigate the link between homophobia and self-esteem in Athens and London, two cities with potentially different levels of perceived homophobia. It investigated the following research questions:

1. Is there a difference in perceived and internalised homophobia between MSM living in London and Athens?
2. Does perceived homophobia relate positively to internalised homophobia and do they both relate negatively to self-esteem of London and Athens MSM?
3. Is there a difference in self-esteem between London and Athens MSM?

The study also attempted to investigate whether societal context is important in explaining the relationship between perceived and internalised homophobia and self-esteem.

## Method

### Participants

The sample included 188 MSM living in London and 173 MSM living in Athens, their mean age being 32 (range 16-64) and 27 (range 16-50) years respectively. The mean time of living in the city was longer for Athens than for London MSM. There was ethnic variation within both samples, but to a lesser degree in Athens than in London; the majority of both samples were White. In terms of sexuality, 89% of London MSM reported that they were sexually attracted to men only, with the remaining 11% being attracted to both men and women. The corresponding figures for Athens MSM were 71% and 29% respectively. In relation to educational level, about 7 in 10 London MSM and 8 in 10 Athens MSM reported to have at least a university degree. Finally, nearly half of the Athenians were recruited and completed the questionnaire online and the rest used a hard copy whereas a small majority of Londoners (56%) filled out the survey online. Table 1 provides a summary of this demographic information.

**Table 1:** Sample characteristics by city

	London (*n* = 188)	Athens (*n* = 173)
Age, years (mean)	32	27

Living in the city, mean	12 years 4 months	20 years 3 months

Ethnic group, %		
White	56.3	86.1
Other	43.7	13.9

Sexuality,^a^ %		
Gay	89.1	71.4
Bisexual	10.9	28.6

Participation method, %		
Offline	44.1	52
Online	55.9	48

a. London *n* = 183, Athens *n* = 168

As there was some scepticism as to whether living in a city for only a few years would be considered sufficient for the participants to be ‘typical’ Londoners or Athenians and because such participants may not have had informed views about how their fellow citizens view gay men in their city, differences in the responses given on all variables of the questionnaire between participants living in London for less than 5 years or more than 5 years were investigated. No statistically significant differences were found. A similar analysis could not be conducted for the Athenian sample because only a handful of participants lived in the city for less than 5 years.

### Procedure

A survey took place in the capital cities of Greece and the UK in Greek and English respectively. Participants were recruited using two methods: the distribution of questionnaires at gay venues (the questionnaires were then self-completed) and through an online questionnaire advertised in several popular gay-themed websites. The study was presented as one examining gay men's views about their social environment, being part of a broader research programme into how gay men see themselves and deal with everyday issues. Potential participants had to be residents of London or Athens for at least the past 12 months. They were assured that their responses and their participation in this research project would remain entirely anonymous and they were informed of their right of withdrawal. Data collection took place over a period of the same 3 months for both cities, although the majority of offline survey data were collected over 2-week periods in each city within those 3 months when the online survey link was live.

### Materials

The study was conducted through a survey that, apart from questions on demographics, included four scales: ‘disclosure of one's sexuality’, ‘perceived homophobia of the general public and of people close to the participants’, MSM's ‘internalised homophobia’, and ‘global self-esteem’. The scales, based on existing English-language scales or developed first in English, were translated to Greek and back-translated until they matched each other so that similar items were asked to both English-speaking participants in London and Greek-speaking participants in Athens. Because of the cross-national element of the study, original scales were subjected to psychometric testing to standardise the scales between the two samples. In particular, all Likert-type scales were subjected to factor analyses for the English and Greek versions separately. Cross-language structurally identical scales were formed following the factor analyses solutions as well as conceptual interpretations, and the reliability of each emerging scale was measured in terms of Cronbach's α in English and Greek separately. Details of the measurements used are given below.

#### Disclosure of one's sexuality

Participants were asked to report the extent to which they were ‘out’ at work, to friends and family. For example, participants had to state whether they had discussed their sexuality with all, some or none of their friends.

#### Perceived homophobia of the general public and of people close to the participants

Participants' perceptions of other people's homophobic feelings and attitudes were assessed using 36 items based on the Modern Homophobia Scale developed by Raja & Stokes.^28^ Eight items were preselected according to their original loadings in the factor analysis that Raja & Stokes ran. Preference was shown to items with higher loadings under each of the three factors of the authors' factor analysis solution as well as items with relevance to this study and its cross-national context. For example, an item referring to gay men being allowed to join the military rather than the item that referred to openly gay celebrities advertising products was chosen despite the lower loading of the former; the reason was that both countries have armies but there are no openly gay celebrities in Greece. Furthermore, the selected items were modified to reflect representations of homophobia; instead of using the original statements written in the first person such as ‘I wouldn't mind going to a party that included gay men’, the statements were modified to read ‘I think that most Londoners/Athenians wouldn't mind going to a party that included gay men’. The same preselected eight items were repeated four times each to capture the perceptions of our participants on how homophobic they think that (a) the general public, (b) their family members, (c) their friends, and (d) their colleagues are. So, the expression ‘most Londoners’ or ‘most Athenians’ was replaced by ‘most members of my family’, ‘most of my friends’, and ‘most of my colleagues’ accordingly. Note that the wording of these representations assessed homophobia in terms of attitudes and behaviour towards gay men only rather than sexual minorities in general. All statements were scored on a 1 to 5 Likert-type scale with 1 meaning ‘strongly disagree’ and 5 meaning ‘strongly agree’. Reliability alphas for the scales made up of 8 items each and assessing perceived homophobia of the 4 different groups of people in both London and Athens were good and varied from 0.76 to 0.93.

#### Internalised homophobia

Mayfield's^29^ Internalized Homonegativity Inventory was used, including its three factors referring to ‘personal homonegativity’ (e.g. ‘I feel ashamed of my homosexuality’), ‘gay affirmation’ (e.g. ‘I believe being gay is an important part of me’) and ‘morality of homosexuality’ (e.g. ‘I believe it is morally wrong for men to be attracted to each other’). Factor analyses run for each sample confirmed the existence of these factors. One item was excluded from the ‘morality of homosexuality’ factor as it was reducing the α of the Greek scale below the 0.60 level; the same item had to be removed from the English version for equivalence. The alphas of the three factors were 0.69, 0.77, 0.90 and 0.63, 0.76, 0.88 for the English and Greek versions respectively.

#### Self-esteem scale

Finally, Rosenberg's^12^ Global Self-Esteem Scale consisting of ten items was used in full to provide a measure of the participants' perception of self-worth. The items of the original scale were rated on a 4-point ‘strongly disagree’ to ‘strongly agree’ scale but we added a middle fifth option of ‘neither disagree nor agree’ to increase variance in the data. A single-factor solution was produced for both city samples with reliability α 0.88 for London and α 0.87 for Athens.

## Results

In analysing the data, missing values met within any section of this survey were not treated in any way, and cases with missing values were excluded analysis by analysis. Because numbers of valid cases for each analysis conducted were adequate, treating of missing values with the potential to affect results was seen as erroneous.

**Fig. 1:**
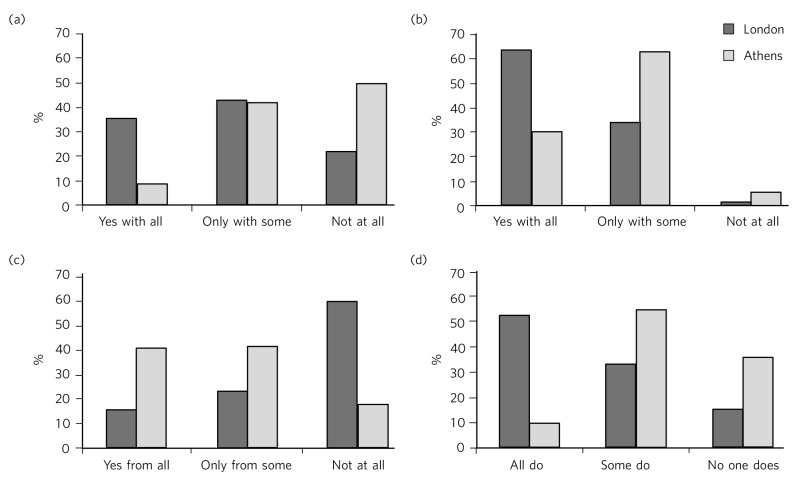
Sexuality disclosure in the study sample. (a) Discussed sexuality with family members; (b) Discussed sexuality with friends; (c) Hiding sexuality from colleagues; (d) Colleagues know about one's sexuality.

Initially, in establishing potential differences in the views and attitudes of Athens and London participants themselves, an important observation was that there were statistically significant differences between the two city groups in relation to the disclosure of their sexuality to other people (Fig. 1). The scores obtained on this measure were subjected to a 3×2 chi-squared analysis. Differences between the samples were found to be highly significant when discussion with family members (χ^2^ = 45.25, d.f. = 2, *P*<0.001) and friends (χ^2^ = 39.15, d.f. = 2, *P*<0.001) was concerned and as far as hiding (χ^2^ = 65.96, d.f. = 2, *P*<0.001) or revealing (χ^2^ = 72.46, d.f. = 2, *P*<0.001) sexuality from colleagues at work or university was concerned.

A multivariate analysis of variance (MANOVA) was then conducted to investigate the differences seen in Table 1 between London and Athens MSM in their views on how they think the general local public and people close to them see gay men (Table 2). Overall, Athens MSM perceived other people's homophobia, whether general public, friends, family members or colleagues, to be higher compared with London MSM perceptions. There was a significant overall difference between the two populations on the combined four dependent variables (*F*_(4, 298)_ = 36.63, *P*<0.001; Wilks's λ = 0.67, partial η^2^ = 0.33). In the separate analyses for each dependent variable, all differences between Londoners and Athenians were also found to be highly statistically significant (Table 3).

**Table 2:** Descriptive statistics of the Likert-type variables of the study

5-point Likert-type variables (*n* items)	London *n* (mean) s.d.	Athens *n* (mean) s.d.
Perceived homophobia		
General public (8)	176 (2.38) 0.59	166 (3.14) 0.63
Friends (8)	176 (1.75) 0.63	156 (2.40) 0.68
Family (8)	175 (2.37) 0.96	156 (3.23) 0.83
Colleagues (8)	173 (1.98) 0.73	155 (2.67) 0.73

Gay men's personal homonegativity (11)	184 (1.87) 0.72	167 (1.95) 0.70

Gay men's negative views on morality of homosexuality (4)	185 (1.25) 0.46	167 (1.37) 0.51

Gay affirmation (7)	186 (3.72) 0.66	166 (3.60) 0.67

Self-esteem (10)	172 (3.92) 0.68	155 (4.10) 0.65

**Table 3:** Between-subjects effects on the variables referring to perceived homophobia of others

Variables	*F*^a^	η_p_^2^
Perceived homophobia of general public (8)	111.889	0.271

Perceived homophobia of friends (8)	85.349	0.221

Perceived homophobia of family (8)	75.872	0.201

Perceived homophobia of colleagues (8)	72.925	0.195

a. d.f. = 1, d.f. for error = 301, *P* = 0.000 for all variables.

In investigating the differences between the two samples in relation to ‘internalised homophobia’ as again seen in Table 1, a one-way between-groups MANOVA showed that there was a narrowly statistically significant difference between Londoners and Athenians on the combined dependent variables (*F*_(3, 340)_ = 2.65, *P*<0.05; Wilks's λ = 0.98, partial η^2^ = 0.02). When the results for the three dependent variables were considered separately, Athens MSM scored significantly higher only on the ‘morality of homosexuality’ variable (*F*_(1, 342)_ = 6.545, *P*<0.05, partial η^2^ = 0.02).

Third, an independent-samples *t*-test was conducted to compare the self-esteem scores (Table 2) for the London and Athens samples. There was a significant difference in scores between the two groups with Athenians reporting a higher self-esteem than Londoners (*t* = −2.421, d.f. = 325, *P*<0.05, r γ λ = 0.13).

Correlational analysis showed, as expected, positive relationships between most perceived and internalised homophobia scales and negative relationships between homophobia and self-esteem scales. Online tables DS1 and DS2 show that these findings are relatively consistent across the two city samples. Further regression analyses were carried out to test the relationship of both internalised and perceived homophobia of others controlling for city. Correlations showed that factors for each scale were significantly associated with each other; this was expected given that they are subscales of the same construct. Therefore, and to avoid multi-collinearity, the composite scores of the scales were used. To test whether city explained the relationship between homophobia (internalised and perceived) and self-esteem, a stepwise hierarchical regression was conducted. The first step tested the relationship between homophobia and self-esteem and the second step tested whether the addition of city had a significant impact on the model. The inter-correlations between ‘self-esteem’ and ‘perceived homophobia of others’ as well as ‘internalised homophobia’ were significant (*r* = −0.15, *P*<0.05 and *r* = −0.27, *P*<0.001 respectively). The correlation between perceived and internalised homophobia was also significant (*r* = 0.29, *P*<0.001). Model 1 is statistically significant (adjusted *R*^2^ = 0.070, *P*<0.001) but self-esteem is explained significantly only by internalised homophobia (Table 4). City, in model 2 has a significant impact on the relationship between internalised and perceived homophobia of others and self-esteem (adjusted *R*^2^ = 0.12, *P*<0.001). In particular, city explains an additional 5% of the model. Moreover, both homophobia scales in the model independently explain self-esteem significantly.

**Table 4:** Explanation of self-esteem by homophobia scales (composite scores) and the role of city context

Independent variables	Standardised β	*T*	*P*⩽
*Step 1*			
Perception of homophobia in others	−0.079	−1.403	n.s.
Internalised homophobia	−0.242	−4.313	~0
F_(2, 321)_ = 13.1, *R* = 0.275, *R*^2^ = 0.076, adjusted *R*^2^ = 0.070, *P*<0.001			

*Step 2*			
Perception of homophobia in others	−0.228	−3.479	0.001
Internalised homophobia	−0.210	−3.805	~0
City context	0.260	4.147	~0
F_(3, 320)_ = 14.9, *R* = 0.351, *R*^2^ = 0.123, adjusted *R*^2^ = 0.12, *P*<0.001.			

## Discussion

The first aim of this study was to explore potential differences between MSM living in Athens and London in relation to how they view their sexuality and on the ways in which others in their broader environment or those close to them see gay men. The findings suggested that there are indeed some significant differences between the samples of the two cities. Athens men were more ‘closeted’ than London men and reported higher levels of homophobia in terms of how the general public and people in their close environment see gay men. However, although Athenians again scored higher in the internalised homophobia scales, such a difference was found to be narrowly significant only when all factors measuring internalised homophobia were combined for the analysis. Finally, there was a difference between scores on the self-esteem scale with Londoners this time reporting lower levels of self-esteem than Athenians. Along these lines, society played a significant role in the relationship between homophobia and self-esteem; the relationship became stronger as a result of city of residence.

In general, Athenians appeared to perceive that they lived in a more homophobic city than Londoners and this could relate to the observation that they felt less comfortable to disclose their sexuality publicly. This finding comes into agreement with the difficulties reported by ethnically Greek gay people in Phellas's^26^ and Fygetakis's^25^ studies. One would expect, however, that Athenians' self-esteem might have been lower than Londoners' self-esteem, which in this study was not the case. This could be due to the likely possibility that sexuality was not considered to be the sole or even the most important and salient element of one's life. Our findings support Abrams & Hogg's^30^ claim that global self-esteem evaluation may not reflect the particular group membership under investigation and also support other findings on the relationship between stigma and self-esteem not being inevitable.^31^ Similarly, Brady & Busse^32^ found no significant difference in terms of psychological well-being and adjustment among open or closeted respondents in the last three stages of Cass's coming out model. Such findings may relate to Alquijay's^33^ argument that, in cultures where interdependence is valued, the meanings of self and self-esteem may be different; this point could be very relevant for our Athens participants. In general, progression through the stages proposed by Cass's model on ‘sexual identity formation’^34-36^ may be influenced by expectations of the Greek society. Global self-esteem and its relationship to stigmatised identity may be negotiated in different ways by Greek sexually stigmatised groups compared with other nationalities or to other type of stigmatised groups, again because of specific societal norms that relate to sexuality.

It needs to be underlined that there was a clear negative correlation between self-esteem and personal homophobia in both samples; this supports findings such as Szymanski *et al*'s,^11^ among others, who linked internalised homophobia to the well-being of lesbians and gay men. There were also significant positive correlations between all measurements of perceived homophobia of family members and colleagues with internalised homophobia variables (the direction was negative for ‘gay affirmation’) and friends' homophobia was found to have the strongest relationship. Such findings demonstrate the potential consequences of societal homophobia on gay men and women. Therefore, it is society that needs to change; this can happen via the promotion of institutional social equity for gay people's self-acceptance and the building of a positive identity as Berg *et al*^8^ argue.

### Limitations

Although our results suggest some interesting relationships, there are limitations regarding the interpretation of findings. One of the main limitations is the correlational nature of the study that does not allow the data to show causal relationships regarding the extent of the contextual impact of homophobia on self-esteem. Additionally, the concepts' measures are related highly to each other and this may have an impact on the results and subsequent conclusions regarding the strength of associations. It would have been beneficial to have used additional outcomes variables that measure mental health and broader well-being. Although self-esteem is a good indicator of well-being, it does not capture its complexity. Therefore, the issue of homophobia and its relationship to well-being in conjunction with societal or cultural differences needs to be unpacked further.

It should also be acknowledged that one of the main limitations of this study is the inability to account for non-response due to the use of online data collection. This is a weakness of internet-based surveys because non-response can threaten the validity of data; participants may differ from non-participants on a number of characteristics. Still, as internet use and internet-based research are gradually becoming more and more common, at least within European contexts such as those in this study, issues of generalisability and validity are dealt with. As Hewson stated,^37^ there is gradually less sample bias as potential internet-based research participants are less and less the White, middle-class, technologically proficient people. Mathy *et al*,^38^ for example, compared the demographics of a small sample of lesbian and bisexual women with a larger sample collected by a large polling organisation and they found that their rigorous internet sampling designs were found to be more robust and equally representative of the US general population. The internet sample was more representative in terms of education and income and broader ethnic diversity and it was equally effective in representing the distribution of population in rural and urban areas. In addition, there is a point to be made on the online facilitation of self-disclosure and this is very relevant for this study as we recruited people who belong to a stigmatised group. The effectiveness of online or computer-based surveys or interviews for researching sensitive issues such as sexual behaviour is well established. There is ample evidence that computerised internet interface tends to facilitate self-disclosure and honesty among research participants and that participants report lower social anxiety and social desirability when they are using the internet than when they are using paper-based methods.^39-45^

In investigating cross-city differences, this study and the way it recruited participants could not have and does not claim to have done such investigations by employing homogeneous cultural groups within each city. Both cities, and especially London, include microcultures within any culture due to the diversity of their populations. This makes it impossible for this study to claim consistency of experiences among participants of each city. However, owing to the way data were collected, the study captures a relatively diverse sample of microcultures that constitute the populations of MSM within each city.

### Relevance to mental health services

In conclusion, until societal changes in terms of homophobia happen, mental health professionals need to be aware of contextual differences in dealing with gay men and women who seek help. Although literature suggests that evidence-based interventions for the general population can also be beneficial for gay men,^46^ studies (mainly qualitative in nature) have shown that LGB services are preferred over mainstream ones.^20^ For example, research has shown that ‘gay affirmative therapy’ is preferred by LGB people as it views LGB lifestyles and sexual identities positively without pathologising them.^47^ However, these findings come from qualitative studies with purposive samples. There needs to be a strong understanding of indigenous psychologies and the relationship between culture and psychology. Providing blanket responses to feelings of rejection, for example, which may encourage clients to disclose their sexuality as part of the process of self-acceptance and building a positive sense of self is not always the optimal strategy. Complete ‘coming out’ should not always be seen as the end goal because it may be that it has much graver consequences than incomplete ‘coming out’ within some societies. The existence of close societal ties between people may be more important for one's well-being. Jeopardising such ties as part of the ‘coming out’ process may be counterproductive for the gay individual. This may be particularly important in societies that do not have a developed gay community and gay movement that could provide alternative adequate support mechanisms.
